# Emerging industry 4.0 and Internet of Things (IoT) technologies in the Ghanaian construction industry: sustainability, implementation challenges, and benefits

**DOI:** 10.1007/s11356-022-24764-1

**Published:** 2022-12-24

**Authors:** Rashid Maqbool, Mohammed Rayan Saiba, Saleha Ashfaq

**Affiliations:** 1 Faculty of Engineering and Environment, Northumbria University, Newcastle Upon Tyne, NE1 8ST UK; 2 School of Economics and Management, Fuzhou University, Fujian, China

**Keywords:** Sustainable construction, Internet of Things, Industry 4.0, Sustainability, Ghana

## Abstract

Future construction projects will need the implementation of industry 4.0 and Internet-of-Things (IoT) technologies. The construction sector has, however, falling behind other industries in the application of these technologies and is currently facing considerable challenges. One of the industries that lag behind in the use of new innovative technological tools is the construction industry. This study reviews the research work in industry 4.0 and the Internet of Things as they relate to construction and examines key Ghana-based construction professionals and firms to ascertain their level of understanding of these emerging innovative technologies, including the challenges and benefits associated with their implementation. An extensive review of pertinent literature was done to help identify the important paradigms and variables which were cautiously tested. Adopting a quantitative research approach, the attained variables were used to design into a close-ended questionnaire. The sample frame was a survey of people from 154 construction experts and researchers with good standing by using the purposive sampling. Relative importance index (RII) analysis was used to analyzed the data. It was discovered from the findings that smart construction was the most popular industry 4.0 technology in the Ghanaian construction industry. The most important benefit of these technologies is that they will add sustainable policy requirements to tendering, with the most pressing technology being the lack of talent and skills in using industry 4.0 and IoT technologies. The scope of this research is based on the questionnaire survey, proving a sustainable pathway to the construction industry community, which creates its own significance by including key stakeholders and those affected by these technologies.

## Introduction

Under the aegis of industry 4.0, innovative technologies can be used to address construction industry issues. Tech-savvy generation Z youths entering the job market are more attracted to technological solutions that automate manual operations and boost on-site and off-site productivity (Ghosh et al. 2020). Today, due to the rapid evolution of technological advancements, devices that are smart can now connect, communicate, and interact via the Internet. Their size has decreased, their computational power and storage capacity have increased. In today’s smart devices, embedded systems can communicate, sense, and act, as well as access, gather, store, and process data in real-time. There are several applications, functions, and services available on the IoT. It is a technology that is gaining traction quickly. The Internet of Things is employed in almost every facet of life and across a wide range of industries. People and gadgets will be able to connect anytime, anywhere, and with anything and everything as a result of the Internet of Things (IoT) pervading our everyday world and its items (Lampropoulos et al. 2019). Kelvin Ashton first introduced it in 1999. It is the ability to connect things via the internet; you can use it to create a platform for performing specific tasks. To carry out any specific function over the network, it makes use of internet connectivity and the surrounding environment. It is a technique for allowing objects to communicate with one another via the internet (Gamil et al. 2020). Technological advances such as the Internet of Things (IoT), big data, and cloud computing have been shown to dramatically improve industrial intelligence, especially in the manufacturing industry. Recently, these technologies have been applied to a variety of sectors of the construction industry to aid in the efficient optimization of design, performance evaluation, and monitoring of project risks as well as energy savings and emission reduction (You and Feng 2020).

Since its inception, industry 4.0 has been well-known around the world, with researchers attempting to determine how the new technology may be used, as well as how industry 4.0 can be sustainable, valid, dependable, and secure. Workers are subjected to new pressures as a result of modern technology. As a result, the construction industry must increase the skills of its current employees (Moshood et al. 2020).

The Internet of Things is now experiencing significant hurdles in the construction industry, including several networks coexisting, enormous data quantities, address restrictions, automatic address configuration, and security tasks such as authentication and encryption. Future construction projects will necessitate the usage of Internet-of-Things (IoT) applications due to their increasing complexity. The construction industry will be left behind by other industries if no application will facilitate the work involved (Mahmud et al. 2018). The construction sector is one of the industries that lag in the adoption of advanced technological digital tools. According to “Made Smarter UK,” construction is one of the industries that could profit from the industry 4.0 revolution (Turner et al. 2020).

The construction sector has a reputation for being slow to adopt new technology and advancements. The industry’s aversion to adopting new technologies stems from several factors, including the perception of exorbitantly high costs associated with new technologies, the training required to implement technological change and innovations, and a desire to avoid disrupting established systems, processes, and procedures (Newman et al. 2020). Whatever the obstacles, the construction industry must improvise and adapt to a constantly changing global economy. Not only does progress connect global developments, world, creating a pre-existing environment but also allows for reversing previous industrial revolutions' impacts on the environment (Maqbool and Wood 2022). Contrary to their benefits, the construction industry has a difficult time adopting IR 4.0 and IoT concepts (Alaloul et al. 2020).

With publications and research focused on smart buildings, construction safety, optimization, and simulation, a small number of professionals are functioning in isolation and offering single-point solutions in IoT and IR 4.0. The application landscape as a whole must be described. The Internet of Things (IoT) has also been investigated in several building scenarios. Despite these constraints, IoT applications and benefits have to be introduced into the construction industry. Despite this, the construction industry’s benefits from IoT applications are still unknown (Dilakshan et al. 2021). The goal of this research is to show how IoT and IR 4.0 technologies can be used in the construction industry by evaluating IoT and IR 4.0 concepts, present applications, difficulties, and solutions.

The aim of the study is to explore the prospects of emerging industry 4.0 technologies and IoT in the construction industry.

1. To identify some emerging industry 4.0 technologies and Internet of Things in the construction industry.
2. To identify the benefits associated with the application of the emerging industry 4.0 technologies and Internet of Things in the construction sector.
3. To identify the challenges facing the construction industry in adopting industry 4.0 technologies.

To achieve the aim and objectives of this study, an extensive review of pertinent literature would be done. It would help to identify the important paradigms and variables which were cautiously tested to achieve the aim and objectives. Adopting a quantitative research approach, the attained variables were used to design into a close-ended questionnaire, i.e., using a quantitative approach to research.

Respondents were mainly the construction experts, from both the academia and industry in Ghana. The sample frame was a survey of people from academia and construction experts with good standing, who are active in the construction industry for at least 5 years and operating within Ghana.

The vital importance of this research is to first bring to light important emerging trends, their application, and impaction in the construction industry. Moreover, it would also delve fill-up the literature of what these technologies and Internet of Things in construction are about. Since every research has an aim and objectives, this would also explore what is needed and what needs to be done to bolster our construction sector with these new technological trends. It would also look at how construction professionals and firms can embrace the Internet of Things and industry 4.0 technologies for efficient project execution.

## Literature review

Ghana’s construction operations include the construction, repair, maintenance, alteration, and demolition of buildings, highways, streets, bridges, roads, sewers, railroads, and communication systems. Given its contribution to GDP, the construction sector continues to assist the expansion of the Ghanaian economy (Ackah et al. 2014). The need for social, utility, and transportation infrastructure has never been greater, affecting the need for affordable housing. As a result of these issues, the construction sector has continued to analyze and redesign itself. This has a favorable influence on the economy since it closes the overall infrastructure deficit (Maskuriy et al. 2019). Thanks to the new emerging technologies, which primarily focuses on the use of computers and cyber-physical systems, major technological improvements have recently been realized in the construction sector. Some of these technologies, such as BIM, sensors, and the Internet of Things, have been shown to be beneficial in attaining the objectives of a sustainable building environment (Forcael et al. 2020). This development has the potential to considerably assist Ghana’s building sector. This technical breakthrough, dubbed “Construction 4.0” at times, has gained appeal in recent years.

Industry 4.0 popularized the concept of the creative industry by using all of the available elements of personalized techniques. The capacity to develop, estimate, and explore new ideas and concepts without disrupting the real world is acceptable in relation to the recently released facts on sustainability (Maqbool and Akubo 2022). However, Ghanaian construction firms are defying this technological progress. As a result of technological advancements, construction workers in Ghana will face new challenges. In order to satisfy corporate needs, construction organizations will need to continue to develop their training of present staff to implement novel approaches. Improving existing workers’ digital skills is a major challenge. The perspectives of the construction industry to accelerate productivity improvements and benefit the industry 4.0 revolution may be compromised since the education system is now attempting to apply the best measurement and kind of skills to match the sector’s requirements (Moshood et al. 2020).

Despite the many benefits of industry 4.0, the Ghanaian construction industry has yet to implement this cutting-edge technology. The construction industry is an important part of the country’s economy. As a result, it is vital to understand the major barriers to implementing new technologies that are specific to construction projects. Industry 4.0 has lately received a lot of attention in the manufacturing industry as a strategy to enhance digitalization and automation utilization with the objective of building a digital value chain for the product’s lifecycle, from idea to development, manufacture, use, maintenance, and disposal. This facilitates the production of high-quality products at a lower cost and shorter time-to-market, as well as an overall increase in company performance (Hossain and Nadeem 2019). Even among construction experts in Ghana and other developing countries, understanding of and use of industry 4.0 and IoT technologies in the construction sector is generally poor. This is due to the adoption of contemporary computing and technology that is still in its early stages (Oke and Arowoiya 2021). The relevance and visibility of adoption of industry 4.0 technologies in companies and industries has increased. As a result, the implications of these technologies for construction sustainability objectives deserve additional consideration and review. The fourth industrial revolution has the potential to solve many of the problems posed by old building processes and technology in order to deliver a more sustainable future (Bai et al. 2020).

### Industry 4.0 and construction industry

Researchers are only just beginning to investigate industry 4.0. In addition to academia, the term is well-known within the industry. Industrial Internet of Things (IoT) is the transformation of how companies connect and control their machinery, factories, and warehouses. This transformation has challenged the industry due to the availability of digital data and online digital access that may be utilized to automatically acquire and process electronic information (Maskuriy et al. 2019). With the adoption of IR 4.0, a new sector will be established in which all automated systems will be integrated through technical improvements to function and communicate information without the use of humans, resulting in increased efficiency. In a smart factory, cloud computing and cognitive computing are utilized to store data and make choices. The Internet of Things (IoT) integrates cyber-physical systems, allowing humans to monitor processes in real time without having to be physically present. As IR 4.0 manifests its visionary capabilities, the Internet of Things (IoT) integrates cyber- physical systems, allowing humans to monitor processes in real time without having to be physically present (Alaloul et al. 2020).

### Application of industry 4.0 primary technologies in the construction industry

Industry 4.0 is defined by advanced automation and digitalization processes, as well as the use of electronics and information technology (IT) in manufacturing and services (Obitko and Jirkovský 2015; Roblek et al. 2016; Yuan 2015). Integration and analysis of massive harmful data in real time will optimize construction resources and result in improved performance. Mobile computing, cloud computing, big data, and the Internet of Things are among Industry 4.0’s most important technologies (Gruber 2013; Roblek et al. 2016; Vijaykumar et al. 2015; Wan et al. 2016). In particular, mobile and cloud computing enable industry 4.0 by integrating industrial IoT networks. Construction 4.0 consists of three subcategories:

- Modelling and simulation,
- Smart construction site (smart manufacturing),
- Digitization.

These technologies have become increasingly common in the last decade due to their rapid adoption in the construction industry. Cloud Computing, BIM, DPD, and RFID are just a few examples. A certain number of emerging technologies (AI, big data analytics, autonomous robotic construction, additive manufacturing, virtual and augmented reality), on the other hand, they are poised to have a major impact on the industry in the coming decades (Koc et al. 2020).

Industry 4.0 aims to modernize the construction industry’s existing management and maintenance systems, as well as to improve the execution of construction projects to smarter and more sustainable levels. IoT, CPSs, and autonomous robotics are some of the significant industry 4.0 technologies that can be used to transform existing construction systems into big data analytics, simulated models, and enhanced re-examination (Lasi et al. 2014). By adapting to changing customer and market demands, industry 4.0 aims to address the competitiveness of today’s construction industry in a dynamic global market. Sustainable construction will pave the way for the economy by integrating cutting-edge technologies into a variety of traditional processes and products (Maqbool et al. 2022b; Zhong et al. 2017). Industry standard 4.0 framework for real-time decision support will include data gathering, analysis, and interpretation from a variety of sources, such as industrial systems and equipment, as well as customer management systems.

The Industrial Internet of Things (IIoT) is a subgroup of the Internet of Things that focuses on its use in advanced and smart industries. The IIoT is considered to be a complicated system made up of numerous industry 4.0 systems and devices. The Industrial Internet of Things (IIoT) for the construction industry, in particular, integrates a variety of critical contemporary technologies to create a system in which the whole is more efficient and sustainable than the parts (Lampropoulos et al. 2019).

IIoT services and applications in construction enable sustainable manufacturing operations and systems’ scheduling, planning, and control. This improves a company’s overall availability and maintenance, as well as its operational efficiency. The IIoT decentralizes analysis and decision-making, allowing for real-time response and reaction via a network of interconnected devices (Gilchrist 2016).

#### Cyber-physical system and the construction industry

As a result of significant technological advancements in the fields of computer science, information technology, and manufacturing, the construction industry has embraced cyber-physical sustainable technologies and frameworks (CPSs) (Mourtzis et al. 2016). The CPS concept enables a cyber sustainable ecosystem in which intelligent machines communicate with one another via a wireless network to manage construction data. Additionally, CPS technologies use complex and unique mechanisms to connect the actual and virtual construction worlds in a seamless manner (Mourtzis et al. 2016). As a result, they are built to provide both physical inputs and outputs, allowing for unique techniques to improve human relationships (Lu 2017).

#### Cloud computing and the construction industry

Cloud computing (Cloud) is a type of outsourcing that uses a vast number of computer servers and resources to provide on-demand, high-level services and resources in real time, and it is crucial to improving and revolutionizing the current construction industry (Bhardwaj et al. 2010). The three service levels of cloud computing are software as a service (SaaS), platform as a service (PAaS), and infrastructure as a service (IaaS), each of which enables virtualization and management of the solution stack at a different level (Bhardwaj et al. 2010). One of the main advantages of the use of cloud computing is its use of advanced applications and services that scale dynamically with the increasing number of users (Lu and Cecil 2016). Users and businesses can also access apps, programs, and services that are promptly delivered and housed in the “cloud” from any location at any time. As a result, industrial such as the construction industry widely utilizes various cloud-based applications, such as customer relationship management (CRM), human resource management (HRM), and others, to enhance their system’s effectiveness. Additionally, construction firms that use cloud computing avoid the costs and difficulties of constructing and maintaining their own IT infrastructure, as well as the upfront expenditures associated with a pay-as-you-go approach, allowing them to start small and scale up as demand for their services grows (Lu and Cecil 2016). Consistent data and service access from any connected device, high availability and maintainability, and cheaper development costs and time to market for the product are just a few of the additional benefits. Given the construction industry’s rapidly changing nature, broad fields of application, and several advantages, big profits can be produced within the industry, and more and more businesses of all kinds and types are increasingly embracing modern technology to expand their capabilities at a cheap cost (Zhong et al. 2017).

#### Advanced data analytics and big data in the construction industry

Intelligent devices and advanced technologies (such as information technology, artificial intelligence (AI), and social networking sites (SNS)) have increased the number of data sources and digital content diversity, as well as the types, forms, and structures of data that are digitized in daily life (Gahi et al. 2016). As a result, a massive amount of heterogeneous data is generated on a daily basis and grows exponentially in size. This is referred to as “Big Data.” The primary characteristics and differences between big data and traditional data are volume, variety, veracity, speed, and value (McAfee and Brynjolfsson 2012).

Big data is significant in sustainable and smart construction as it allows organizations to use predictive and prescriptive analytics to get a variety of advantages, merits, and benefits. As a result, construction firms that want to stay competitive should make advanced analytical tools, techniques, processes, and apps a priority for processing enormous amounts of data, extracting insight, and recovering the value of important data in each case. Big data analytics is a set of tools that investigate vast volumes of diverse, quickly changing data using contemporaneous and analytic methodologies, enabling for the collection, processing, and management of critical data and statistics (Parwez et al. 2017). By far, the most effective sustainable approach for companies to ensure a substantial competitive edge is to use newly acquired data to develop important insights, improve equipment care and maintenance, optimize operations, and boost productivity, quality, and efficiency (Gilchrist 2016). To fully utilize all of big data’s benefits, however, businesses must alter their decision-making culture and recognize the importance of human insight, regardless of how great the significance of big data and analytical techniques becomes (McAfee and Brynjolfsson 2012).

### Challenges faced in implementing industry 4.0 technologies

VAIDYA et al. (2018) defined several challenges and fundamental issues in several sections of the industry 4.0 implementation. In their research, they identified sections which were:

- The smart decision-making mechanisms and negotiations.
- Protocols for high-speed industry wireless Internet (IWN),
- The production and analytical production of specific big data.
- The design and analysis of systems.
- Cyber-safety and property protection.
- The modular and flexible use of physical devices.
- Issues of investment

Smart construction, network building, big data analysis, digital production, and processing are some of the scientific and technology problems associated with industry 4.0 deployment in the construction sector (Zhou et al. 2015). A lack of a digital strategy aligned with resource constraints and a lack of standards and inadequate data security are major impediments to industry 4.0’s technological implementation (Schröder 2016). Due to various concerns and barriers, the construction industry is reluctant to implement industry 4.0. Küsters et al. (2017) added that uncertainty about financial gains, a lack of strategy coordination across multiple organizational units, a lack of talent and skills, a reluctance to make radical changes, and concerns about the security of third- party suppliers are among these concerns. The three major challenges associated with industry 4.0 implementation in the construction sector, according to Kagermann et al. (2013), are (1) standardization, (2) work organization, and (3) product availability. Smart equipment requirements, deep connectivity networks, and know-how-driven manufacturing continue to face obstacles and challenges (Chen et al. 2017). Interoperability was identified as the primary unresolved issue in industry 4.0 by Lu (2017), who investigated the key principles for ensuring process precision and efficiency, such as accessibility, security, privacy, multilateral solutions subsidiarity, open-source software, and multilingualism.

### Internet of Things (IoT) in construction industry

IoT is defined as a system of “Things” that connect and communicate with each other over the Internet or a private network. Intelligent devices that are networked and communicate with each other with minimal human involvement are defined as “things” (Dilakshan et al. 2021). IoT can be divided into three layers based on its conceptual definition: the sensing layer the application layers and the network layers (Chen et al. 2020). The target’s sensing layer, which consists of perception nodes and perception networks, is responsible for sensing and collecting data. It is the network layer that is responsible for data transportation, making it the most important layer. Application layer: This is the top level of the application stack, where end-users interact with it, receiving and presenting the data to users for further services (Jia et al. 2019).

As of today, IoT applications in construction are dispersed, and connectivity barriers exist due to the remoteness of sites and work locations. Construction-specific IoT frameworks seem necessary in light of IoT’s revolutionary potential. To ensure a smooth implementation, it is necessary to address the shortcomings caused by the lack of connectivity standards within that framework.

In the construction industry, it is predicted that the Internet of Things will save 22–29% of total costs, equating to US $75–96 billion in annual benefits (Ghosh et al. 2020). The Internet of Things (IoT) will provide quick reporting while also lowering communication costs and potentially eliminating human error or omissions. In addition, enhanced algorithms and artificial intelligence that can interpret data rather than just analyze it can help with process control and optimization. Researchers in the construction industry are attempting to harness the IoT’s various potential benefits, which are becoming increasingly prevalent. Better accountability, transparency, and monitoring would be possible as a result of the massive amount of data gathered at the micro-level (Ghosh et al. 2020).

### Internet of Things in the construction sector — a need for future applications

The Internet of Things, according to the evidence, has a wide range of potential applications in the construction industry (Tang et al. 2019), allowing unprecedented data volumes to be collected, recorded, handled, and synthesized into useful insight (Kobusińska et al. 2018). As a result, IoT adoption in the construction sector can create additional economic opportunities and contribute to a larger data ecosystem, making future data-driven insights more accessible (Louis and Dunston 2018; Bilal et al. 2016). Integration of the Internet of Things with building information modeling (BIM) to create digital twins enables the development of applications that improve construction and operational efficiency (Shahzad et al. 2022). Furthermore, the Internet of Things (IoT) is viewed as a catalyst for cyber-physical construction, also known as construction4.0 (Gamil et al. 2020; Sawhney et al. 2020).

The IoT presents practitioners with impressive opportunities to enhance the construction industry’s reputation and take the lead in resolving its time and resource constraints through the use of advanced technologies. Construction sites are usually scattered across broad areas, demanding remote coordination with many departments and resources to guarantee comprehensive, quick, and automated workplace decision-making (Louis and Dunston 2018). The Internet of Things makes use of technological innovations like sensors and linked devices to monitor real-time characteristics as well as techniques like large-scale data processing and data mining to provide esthetically appealing end results (Riaz et al. 2006, 2017). In order to address modern technological challenges in this industry 4.0 era, the construction industry must adapt and transform from primitive methods to digitalized automated processes as a critical step toward increasing productivity, efficiency, and environmental sustainability, as well as dynamic management and planning (Maqbool et al. 2022a; Dallasega 2018). The Internet of Things is expected to significantly impact the construction sector financially by ensuring high reporting speeds and lowering communication costs (Ramasundara et al. 2018) and by improving process control and optimization (Madakam and Uchiya 2019). Even at the micro level, the massive volume of big data collected will improve monitoring and analysis, increase accountability and transparency, and highlight the adequacy of key performance indicators (KPIs). Sustainable construction processes and methods will be altered significantly if cutting-edge technologies based on IoT, and the large computational capability of cloud-based servers are implemented. The core technologies that will revolutionize the construction industry are robotic bricklaying (FastBrick Robotics), automated OH&S reporting, and integrated technology into construction components that provide intelligent structural components (AutoDesk Fusion Connect) (Smart Products). These technologies rely on the Internet of Things and massive amounts of computing power from network sensors to monitor and control these operations (Edwards et al. 2017).

### IoT and industry 4.O in sustainable construction

In this digital age, sustainability is both a pressing demand and a technical problem. The development of smart technologies is important to ensure the sustainability of future industrial systems. Much research has been conducted on IoT-enabled sustainable production in industry 4.0 from the technological, commercial, organizational, and operational perspectives (Leng et al. 2020). These industry 4.0 technologies have the potential to greatly increase competitiveness and innovation while also maintaining the long-term sustainability of the current industrial framework (Müller et al. 2018). In the future, industry 4.0 will play a significant role in more sustainable industrial value development (Stock and Seliger 2016).

Initially, economic sustainability is the major objective of sustainable production. Construction that is environmentally friendly is also critical for global society’s long-term growth (Maqbool and Amaechi 2022). The populated connectivity of socialized production resources and open-architecture products is the fundamental trend of the aforementioned sustainable construction ideas (Ghobakhloo 2018).

The IoT will play a significant role in sustainable construction, allowing for real-time data collection on energy use (Kara et al. 2011). According to Haller et al. (2008), this data must then be incorporated into production management processes and choices to increase energy efficiency. Autonomous technologies, such as the Internet of Things (IoT), are converting the construction industry’s surroundings into smart factories. Green buildings are those that are built with the least amount of energy possible, not only those that consume less energy when in use (Maqbool et al. 2020). Escalating energy prices, increased environmental consciousness, and changing customer behavior are putting pressure on decision-makers to prioritize green manufacturing and energy-efficient production practices (Maqbool and Jowett 2022; Shrouf et al. 2014).

Digitalization and sustainability are cross-cutting topics in all stages of the production process. Industry 4.0 is projected to have a positive impact on productivity, flexibility, and resource efficiency (e.g., big data for predictive maintenance). Future technologies include closed-loop construction systems connecting machines, information systems, and products and stakeholder engagement/collaboration (Waibel et al. 2017). Sustainability is frequently considered in the construction industry in terms of the environment, society, and economy (Maqbool et al. 2022c).

Industry 4.0 new technologies include IoT, 3D printing, robotic systems, cloud computing, virtual reality, simulation, prototyping, and more. Big data is characterized by a huge volume, velocity, and diversity of data, which necessitates the employment of specialized technology and analytical methods to convert it into valuable information. Cloud computing is one of the technologies used for storing enormous amounts of real-time data acquired from numerous sources. In industry 4.0, cyber-security and cloud computing are working together to improve cybersecurity. It is critical to link physical systems to the cloud for system optimization, rapid decision-making, and quality management (Rane et al. 2019). Unlike conventional processes, 3DP technology has been shown to be a cost-effective strategy in architecture, engineering, and construction (AEC). Some traditional mechanisms’ tasks, like concrete mixing, building blocks, and labor, can be substituted by 3DP technology (Singh et al. 2021).

Certain attempts have been made to integrate ecological sustainability with industry 4.0, but certain shortcomings remain. Smart deliveries and coordination can be simulated to decrease the environmental effect of last-mile delivery, lowering CO2 emissions. With sensors and other industry 4.0 technology, simulation may be used to anticipate air quality, noise, and waste products (Gunal 2019).

Adoption of industry 4.0 could help the Ghanaian construction industry. Material prices can be reduced by limiting defective items, and procedures can be improved in terms of yield or speed. Improving production processes, such as optimizing material use, can boost profits and increase productivity. People are still arranging benefit production in the 4th industrial revolution. The implementation of industry 4.0 technologies in the construction industry extends the employment term and encourages the use of contemporary construction and manufacturing instruments through ecosystem principles. As a result, this can contribute significantly to the environmental and economic aspects of sustainability (Moshood et al. 2020).

Nonetheless, the demands for innovative techniques are increasing, and a broad skill set is required. To achieve industry 4.0 in construction, new knowledge and information technologies must be connected in a sustainable manner. Surplus inventory operators in Ghana’s construction sector might overcome barriers such as an unexpected market strategy and excess stock by implementing industry 4.0 technology. Industry 4.0 has opened up a world of possibilities for achieving sustainable production. Organizations should take advantage of possibilities to allow product re-use and remanufacture (Manavalan and Jayakrishna 2019). One of the issues in a developing economy is that demand for industrial systems is increasing, yet companies must also focus on sustainability (Bocken et al. 2014).

### Challenges in implementing IoT in sustainable construction

In terms of new technology, a low acceptance rate remains a challenge due to a variety of factors and this makes construction players hesitant to use IoT in their projects. The biggest obstacle is the scarcity of experts who understand how to use these devices effectively, because becoming an expert at using the equipment necessitates a significant amount of knowledge and training (Salleh and Fung 2014). Furthermore, the construction organizations will incur additional costs in order to train its employees on how to use these devices. Construction companies would find it challenging to embrace IoT due to the high cost of equipment procurement and ongoing maintenance (Salleh and Fung 2014). When the device is operated, the Internet connection will be the primary means of connecting the IoT device to the data transfer system (Mimos 2015). Device interoperability is disabled and the operator is unable to receive data if the Internet connection is poor or unavailable (GSM Association 2014). This means that the construction site requires a strong internet connection in order to operate smoothly and to utilize the best technology feasible to assure data accuracy and interoperability (Mimos 2015). Another impediment to IoT adoption is a lack of awareness among construction workers (Rad and Ahmada 2017), which is especially acute for small and medium-sized businesses due to their limited exposure to the global construction and technology industries (Ahmad Zaidi 2017). On the other hand, they are averse to change, as technology evolves at a constant rate, necessitating constant updating and modification, wasting time, and incurring additional costs when IoT devices are modified (Ibrahim et al. 2019). As a result, despite being aware of the benefits of IoT in terms of saving time, money, and improving project performance, many construction players continue to use traditional methods (Brous et al. 2020). Another issue arises when the government and organizations are unable to persuade construction industry players to adopt IoT (Rad and Ahmada 2017). Government regulation is critical to promoting IoT adoption in the construction industry by establishing standards for IoT use on construction sites.

This can be accomplished by requiring them to operate their project using building information modeling (BIM), cloud computing, big data, sensors, and remote operation tools.

### Overcoming industry 4.0 and IoT challenges in the construction industry

A recent Boston Consulting Group report concludes that the whole digitalization process in non-residential construction will save from 13 to 21% in the engineering and building phases and 10 to 17% in the operational phases annually within the next ten years (BCG 2016). Other developments are considered technological motivators and enablers. The rapid innovation of each construction technology is one enabler.

Lowering construction costs and increasing functionality and improving construction sustainability have resulted from advancements in the Internet of Things and the Internet of Services. Sensors that were previously prohibitively expensive to build and operate have become more affordable. Furthermore, Cloud computing using advanced 5G network infrastructure has enabled a much broader range of audiences to gain access to high-performance computing. On the other hand, BIM is emerging as a platform that integrates all of the 4.0 technologies associated with the creation of semantically digital buildings, both individually and collectively. Although slowly, the subsequent waves of BIM acceptance paved the way for the digitization of various types of constructional information, affecting virtually all project phases (design, construction, and operation).

Through integrated project delivery (IPD), it promises to begin addressing vertical (fragmentation between the life cycle, construction, and business phases) and horizontal (fragmentation across disciplines such as electricity, mechanics, and so on) problems in a much more collaborative manner. IPD is a project implementation method that incorporates people, businesses, and systems, as well as structures and practices, into a process that employs all participants’ talents and insights to reduce waste and improve efficiency throughout all stages of design and construction (Lahdenperä 2012). Overall, increased technology availability and accessibility are creating an innovation pull as well as an understanding of the need for better cooperation in project delivery.

## Research methodology

### Research design

Research design is a broad strategy for acquiring data on a research issue. It is a method for conducting research that answers a specific research issue and involves at least three steps: data collection, instrument creation, and sampling procedures (Bhattacherjee 2012). According to Leedy and Ormrod (2005), a descriptive survey involves asking people questions and tabulating their responses to learn more about one or more groups of people. Leedy and Ormrod (2005) in their study also explained that the primary purpose of survey research is to understand a large population by surveying a representative sample and then summarizing the results using statistical tools.

### Research procedure

This study began with an exhaustive literature search on existing work on industry 4.0 practices in public-funded initiatives, followed by a survey employing a face-to-face and postal questionnaire technique. The collected data is then analyzed statistically.

1. Search for literature about industry 4.0 practices in libraries and on the internet, including journals, publications, research theses, and relevant textbooks.
2. A questionnaire survey applying sampling methods to quantity surveying, architectural, and engineering firms registered with their mother institution in Ghana.
3. The use of a computer statistical package for social sciences (SPSS) version 2.0 to analyze the questionnaire data using statistical techniques

### Target population

A population is a collection of people, objects, or things that are counted (Saunders et al. 2009). Borg and Gall (2009) describe a target population as “a universal set of research of all members of a real or hypothetical collection of people, events, or objects to which an investigator seeks to generalise the finding.” The population of the research includes architects, engineers, construction managers, and quantity surveyors who are registered with their parent institutions, such as the Ghana Institution of Surveyors (GhIS) and the Ghana Institution of Engineers (GhIE) and practise within Kumasi. The sample size was determined using 154 professionals.

### Sample selection

The study used non-probability sampling which is the purposive sampling technique as a sampling technique. According to Parker et al. (2019), purposive sampling is virtually synonymous with qualitative research. The goal of the sampling was to obtain information about a population by watching a small fraction of it. When choosing a sample, all sampling units should be listed or compiled to produce an accurate sample frame, from which the sample size is then chosen. An initial survey was conducted from which, construction industry players were selected from academia and operating construction firms in Ghana.

### Sample size

The term “sample” refers to a portion of a larger group (population) drawn to represent the remainder (Naoum 2012). The study’s purpose, population size, the risk of accepting a “poor” sample, and the permitted sample error are all factors to consider when deciding sample size (Israel and Richter 2011). He also elaborated that a confidence level of 95% and a precision level of 5% are used by researchers. In this study, the researcher took responses from both the construction firms (firms perspective) and the individual professionals in the construction industry (individual perspective). After establishing the sampling frame for this investigation, the researchers adopted the purposive sampling technique to collect data, since the data was collected from construction professionals with respect to their overall knowledge and experience of construction projects rather than any specific projects. This way a potential biased opinion is also avoided in the data.

### Characteristics of the sample

An acceptable sample size from the population being sampled, according to Lemeshow and Taber (1991), should contain the following characteristics:

A list of individuals of the defined population should be included in the size.

i. The population size should be a complete, up-to-date list.
ii. Because no members of the population are listed more than once, this argues that no population member should be listed more than once.
iii. The list should include personal information about each person that can be used to stratify the sample.

#### Sources of data

Data was acquired for the study from both primary and secondary sources. During the survey, primary data was acquired directly from respondents. The main data provided the study with accurate and dependable information from first-hand sources. The library, the internet, diary articles, daily newspapers, and research reports were used to obtain secondary data. The goal of secondary data is to gather the necessary information to direct the study’s execution, with the ultimate goal of validating or refuting primary data.

#### Questionnaire development

The questionnaire was designed and structured to help the researchers achieve their research aim and objectives. The survey was divided into four sections: A, B, C, and D. Part A asked respondents to submit demographic information such as their professional credentials, highest degree of education, and years of work experience. Part B asked respondents to rate their familiarity with various emerging industry 4.0 and IoT technologies mentioned in the literature study. These were to be scored on a scale of 1 to 5. Part C asked respondents to rank some variables related to the benefits of IoT in the construction sector on a Likert scale of 1 to 5. The benefits were described in full in the literature review. On a scale of 1 to 5, respondents were asked to rank industry 4.0 and IoT challenges revealed in the literature review in section D. The enquiries were designed to help the study’s purpose be met.

Most of the questionnaires were personally distributed and few used the addresses obtained from institutes and institutions, and others were sent by email. The distribution of questionnaire and gathering of responses was done over a period of 2 months.

### Data analysis techniques

The data was collected using closed-ended questionnaires. The information received will assist us in accomplishing the study’s goal. There are two parts covered in data analysis section. In the frequency Table 1, the respondent profile is evaluated using the descriptive statics tool of analysis from SPSS Window version 20. The study’s objectives are discussed in part two. Basically, the level of awareness, benefits, and challenges associated with industry 4.0 and IoT in the construction industry. The relative importance index (RII) analysis was used to analyze them.

**Table 1:** Highest level of educational qualification and experience

Variables	Category	Frequency	Percent (%)
Highest educational qualification	PHD	23	15.0
	Masters	26	17.0
	First degree	82	53.0
	Diploma	18	12.0
	Others	5	3.0
Years of professional experience	1–5 years	73	47.0
	6–10 years	35	20.0
	11–16 years	25	19.0
	Over 16 years	21	14.0

## Analysis of data and discussion of results

This section entails analyses of data obtained through circulated questionnaires, as well as a discussion of the outcomes of the data analysis using the Statistical Package for Social Sciences (SPSS version 26). The questionnaire included closed-ended questions that were used to collect data. The information acquired from the data gives the necessary information to meet the goal of this research project. The collected data were analyzed using the one-sample *T*-test and mean score ranking. Other tests, such as the Cronbach’s alpha coefficient test, were also performed to ensure the internal consistency of the test scales used.

### Analyses of demographic profile of respondents

This section summarized the data analysis and discussion of the respondents’ demographic information. Its purpose was to investigate the respondents’ backgrounds in order to validate the dependability of their responses in addressing the objectives of this research. Knowing the respondents’ backgrounds contributes to the credibility and dependability of the data gathered (Pandey and Pandey 2015). Respondent’s professional background, years of experience, and highest educational qualification were all gained.

Top specialists from registered construction firms received the questionnaires. Due to this distribution, we were able to obtain correct responses for our analysis. After sending out 160 questionnaires as our sample size, were returned, resulting in 154 construction professionals responding out of a total of 154 construction professionals considered. Despite this, not all of the surveys were given out and collected in person. As a result, 96% response rate from the targeted 160 professionals was received. Most academic research addressing top management or organizations representatives, according to Baruch (1999), requires a response rate of around 35%. This demonstrates that a 96% response rate is acceptable. Furthermore, the obtained response rate was comparable to that of Essel et al. (2009), who had a 53.7% response rate, and Ahadzie (2007), who had a 45.0% response rate, indicating that the response rate for this study was adequate.

#### Year of experience

This section sought to gather data on the years of experience of the respondents in their above stated professional backgrounds. Drawing from Table 1, the majority of the respondents fell under the category of between 0 and 5 years that is sixty-six (73) out of the total ninety-one (154) respondents represent 47.0%, twelve (35) respondents representing 20.0% fall under 6–10 years, five (25) respondents representing 19.0% fall under 11–15 years, and eight (21) of the respondents had over 16 years of experience representing 14.0%. A diverse experience, according to Leksakundilok (2004), ensures that they can represent the community, which in this case is the construction community.

#### Highest educational qualification of respondents

Respondents were asked to indicate their highest level of educational qualification as part of determining their capacity and credibility to understand the survey. Table 1 presents data collected from respondents on their highest educational level.

From Table 1, it can be deduced that twenty-four (23) respondents representing 15.0% have had a PhD degree, fifty-three (26) respondents representing 17.0% have a master’s degree, eighty-two (82) respondents representing (53.0%) had a bachelor’s degree and fourteen (18) and five (5) respondents representing 12.0% and 3% had a diploma and other certificates respectively. As can be determined from the data, the majority of the respondents hold a bachelor’s degree. Most respondents had a bachelor’s degree, as may be inferred. According to Hegarty (2011)’s research, academic qualifications can assist in gaining additional information for professional growth and organizational development. The mean score ranking and RII of emerging industry 4.0 and IoT technologies are presented in Table 2.

**Table 2:** Mean score ranking and RII of emerging industry 4.O and IoT technologies

Technology	(1)	(2)	(3)	(4)	(5)	RII	Rank
Smart construction sites	35	58	33	22	6	0.7221	**1st**
Big data and advanced data analytics	34	53	38	24	5	0.7130	**2nd**
Building information modelling (BIM)	33	51	35	29	6	0.6987	**3rd**
Cloud computing	23	53	38	24	16	0.6558	**4th**
Simulation and modelling	32	39	38	27	18	0.6519	**5th**
Digital project delivery	29	39	41	31	14	0.6494	**6th**
Mobile computing	28	44	36	28	18	0.6468	**7th**
Autonomous robotic construction	25	38	47	35	9	0.6455	**8th**
Virtual models	25	47	31	33	18	0.6364	**9th**
Cyber-physical systems	21	40	49	27	17	0.6273	**10th**

### Awareness level of emerging industry 4.O and IoT technologies

Industry 4.0 aims to modernize and improve existing manufacturing facilities, management and maintenance systems, and technology by incorporating relevant technologies such as the Internet of Things, Cloud Platform Services, autonomous and flexible collaborative robotics, simultaneous data use and real-world reflectance into a virtual model, big data analytics, and enhanced re-examination (Pereira and Romero 2017). Its goal is to respond to changing customer and market expectations by addressing the global market’s dynamic character and the competitiveness of today’s industries. On a scale of 1 to 5, respondents were asked to rate their familiarity with the following current emerging industry 4.0 and IoT technologies where 1 — extremely aware; 2 — very aware; 3 — somewhat aware; 4 — not so aware; 5 — not at all aware. A mean score ranking and relative importance index (RII) analysis were used to evaluate the awareness of emerging industry 4.0 and IoT technologies. For a scale of 1–5, a mean equal to the hypothesized mean of 2.5 or more means the variable is important. When two or more variables have the same means, the highest-ranking is assigned to the one with the least standard deviation (Field, 2005). The results from the analysis are shown in Table 3. However, before analysis, the reliability and validity of the data were checked to enhance the quality of the data collected. Cronbach’s alpha was used to check the internal consistency of the test scale which is expressed as values between 0 and 1. As established by Tavakol and Dennick (2011), validating the reliability of a measuring instrument is indispensable for evaluation in research studies. They further stressed that a scale is considered reliable if Cronbach’s alpha test results in a co-efficient of 0.700 or greater. From Table 2, Cronbach’s alpha coefficient is 0.945 which falls within the reliability requirement. Hence, the scale used is reliable.

**Table 3:** Reliability analysis

Reliability statistics	
Cronbach’s alpha	**No. of items**
0.945	10

The most popular emerging industry 4.O and IoT technology with regard to respondent’s awareness is “Smart construction” with a relative importance index (RII) of 0.7221. This was followed by “Big data and Advanced Data Analytics” (*RII* = 0.7130), “Building Information Modelling (BIM)” (*RII* = 0.6987), “Cloud Computing” (*RII* = 0.6558), “Simulation and Modelling” (*RII* = 0.6519), “Digital project delivery” (*RII* = 0.6494), “Mobile Computing” (*RII* = 0.6468), “Autonomous Robotic Construction” (*RII* = 0.6455), “Virtual Modelling” (*RII* = 0.6364), and “Cyber-physical systems” (*RII* = 0.6273) were the ranked variables, ranking first, second, third, fourth, fifth, sixth, seventh, eighth, nineth, and tenth respectively.

Industry 4.O attempts to address the turbulent international market and the competitiveness of today’s industries in response to constantly changing consumer and market expectations. The horizontal and vertical system integration will allow the creation of a network of capabilities, functions, Departments, and businesses that will allow for the automation of the value chain. As cutting-edge technology is applied to a variety of traditional items and processes, smart manufacturing will pave the way for today’s industry and economy (Zhong et al. 2017). The mean score ranking and RII of benefits associated with the application of the emerging industry 4.0 and IoT technologies are presented in Table 4.

**Table 4:** RII of benefits associated with the application of the emerging industry 4.0 technologies and Internet of Things in the construction sector

Benefits	(1)	(2)	(3)	(4)	(5)	RII	Rank
Adding sustainable policies as a requirement in tendering	32	35	38	30	19	0.5597	**1st**
Economic benefit	30	43	29	35	17	0.5558	**2nd**
Maintenance of machinery and equipment promptly	30	44	33	28	19	0.5506	**3rd**
Improved project handling with minimum human effort	28	47	34	25	20	0.5506	**3rd**
Human resource management	30	49	28	26	20	0.5416	**4th**
Minimized project delay by taking preventive measures	36	36	39	26	17	0.5377	**5th**
Up-to-date information for better decision making	33	47	27	29	18	0.5377	**5th**
New opportunities and solutions	32	44	37	24	17	0.5351	**6th**
Remote operation of activities on worksites	39	44	29	26	16	0.5169	**7th**
Identification of flaws in construction phases	45	43	24	25	17	0.5039	**8th**

### Benefits associated with the application of the emerging industry 4.0 technologies and Internet of Things in the construction sector

Collaboration between big contractors and low-level suppliers has a significant impact on the value chain of the construction industry. Furthermore, construction projects are locally based and complicated, necessitating significantly greater management (Oesterreich and Teuteberg 2016). The growth of the Internet of Things has a considerable influence in this context and provides various benefits to projects and participants. Similar to objective 1, respondents were also asked to indicate the benefits associated with the application of IoT and industry 4.0 technologies to which they agree or disagree with on a scale of 1–5 where 1 — strongly disagree; 2 — disagree; 3 — undecided; 4 — agree; 5 — strongly agree. Mean score ranking and relative importance index (RII) were performed to assess the benefits associated with the application of the emerging industry 4.0 technologies and Internet of Things in the construction sector. For a scale of 1–5, a mean equal to the hypothesized mean of 2.5 or more means the variable is important. The results from the analysis are shown in Table 5. Similarly, before analysis, the reliability and validity of the data were checked to enhance the quality of the data collected.

**Table 5:** Reliability analysis for objective 2

Reliability statistics	
Cronbach’s alpha	**No. of items**
0.963	10

From Table 4, the Cronbach’s alpha coefficient is 0.963 which falls within the reliability requirement. Hence, the scale used is reliable.

The most important ranked benefit associated with the application of the emerging industry 4.0 technologies and Internet of Things in the construction sector as agreed by the respondents is “Adding sustainable policies as a requirement in tendering” with a relative importance index (RII) of 0.5597. This was followed by “Economic benefit” (*RII* = 0.5558), “Maintenance of machinery and equipment” and “Improved project handling with minimum effort” (*RII* = 0.5506), “Human resource management” (*RII* = 0.5416), “Minimized project delay by taking preventive measures” and “Up-to-date information for better decision making” (*RII* = 0.5377), “Remote operation of activities on worksites” (*RII* = 0.788), “New opportunities and solutions” (*RII* = 0.5351), “Remote operation of activities on worksites” (*RII* = 0.5169), and “Identification of flaws in construction phases” (*RII* = 0.5039) were the ranked variables, ranking first, second, third, fourth, fifth, sixth, seventh, and eighth respectively.

### Challenges associated with the industry 4.0 and IoT implementation

On a scale of 1 to 5, respondents were asked to rate how serious they thought the following challenges were related to the implementation of industry 4.0 and IoT where 1 — strongly disagree; 2 — disagree; 3 — undecided; 4 — agree; 5 — strongly agree. The acquired data were analyzed using the one-sample *t*-test. The one-sample *t*-test is used to measure the relative importance of variables (Ahadzie 2007). A one-sample *t*-test with an accepted hypothesized mean of 2.5 (Uo) shows that concerns with a mean of 2.5 or higher are severe challenges in the implementation of industry 4.0 and IoT. The degree of confidence was established at 95%, which is in line with previous research by (Owusu-Manu et al. 2020). The degree of confidence was established at 95%, which is in line with previous research by (Owusu-Manu et al. 2020). The null hypothesis (Ho) in this study is that “the mean value is not statistically a pressing challenge associated with the industry 4.0 and IoT implementation,” and the alternative hypothesis (Ha) means that “the mean value is pressing challenge associated with the industry 4.0 and IoT implementation.” Before the analysis was conducted, the reliability and validity of the data were checked to enhance the quality of the data collected. If the Cronbach’s alpha test yields a co-efficient of 0.700 or higher, the scale is considered reliable (Tavakol and Dennick 2011). From Table 6, the Cronbach’s alpha coefficient is 0.957 which falls within the reliability requirement. Hence, the scale used is reliable.

**Table 6:** Reliability analysis

Reliability statistics	
Cronbach’s alpha	**No. of items**
0.957	10

From Table 7, a lack of talent and skills in using IoT devices is the most pressing challenge associated with industry 4.0 and IoT implementation with an RII of 0.5247. Lack of awareness among construction workers was ranked by the respondents as the 2nd most pressing challenge associated with the industry 4.0 and IoT implementation with an RII of 0.5197. Concerns about security, hesitation to undergo radical changes, uncertainties about financial gains, open-source software and multilateral solutions, extra cost of training employees in using IoT devices, high cost of acquiring equipment and regular maintenance and multilingualism, and lack of coordination strategies across various organizational units were ranked as the 3rd, 4th, 5th, 6th, 7th, 8th, and 9th most pressing challenges associated with the industry 4.0 and IoT implementation, each with RII of 0.5182,0.5143, 0.5078, 0.5013, 0.4974, 0.4922, and 0.4610 respectively.

**Table 7:** Mean score ranking and RII of challenges associated with the industry 4.0 and IoT implementation

Challenges	(1)	(2)	(3)	(4)	(5)	RII	Rank
Lack of talent and skills	24	64	28	22	16	0.5247	**1st**
Lack of awareness among construction workers	40	43	26	29	16	0.5195	**2nd**
Concerns about security	44	46	18	21	25	0.5182	**3rd**
Hesitation to undergo radical changes	39	47	23	31	14	0.5143	**4th**
Uncertainties about financial gains	42	42	30	25	15	0.5078	**5th**
Open-source software and multilateral solutions	39	52	23	26	14	0.5013	**6th**
The extra cost of training employees in using IoT devices	36	55	28	22	13	0.4974	**7th**
High cost of acquiring equipment and regular maintenance	42	51	23	24	14	0.4922	**8th**
Multilingualism	47	46	22	21	18	0.4922	**8th**
Lack of coordination strategies across various organizational units	45	52	28	23	6	0.4610	**9th**

In this section, the survey data was strategically analyzed using a variety of methodologies of analysis that were best suited to the survey at hand (whether it is showing differences or relationships between variables or groups). Using frequency tables and graphs, the respondent profile was initially evaluated. The first objective was to identify some of the construction industry’s emerging industry 4.0 technologies and the Internet of Things. The Cronbach’s alpha coefficient was used to examine the scale’s reliability, and the relative importance index (RII) was used to measure the level of awareness of emerging industry 4.0 technologies and the Internet of Things in the construction sector. The second objective, to identify the benefits of implementing emerging industry 4.0 technologies and the Internet of Things in the construction sector, was also explored using RII, as well as Cronbach’s alpha coefficient for reliability. For the third and final objective, the relevance of identifying the challenges confronting the construction sector in implementing industry 4.0 technology was also determined using RII analysis.

In order to raise awareness, the study identified some emerging IoT and industry 4.0 technologies in the construction industry. It exhibited the most widely used industry 4.0 and IoT technologies in the construction industry. The most common emerging industry 4.0 and IoT technologies, according to the findings, are smart construction, big data and advanced data analytics, and building information modelling (BIM). The study also highlighted that the lesser-known industry 4.0 and IoT technologies are virtual models and simulation and modelling cyber-physical systems. Efforts should be made to improve knowledge of industry 4.0 and IoT technologies in the construction sector, with the less well-known technologies necessitating a greater investment.

According to the findings, the most significant benefits associated with the application of industry 4.0 and IoT technologies are the addition sustainable policy requirement to tendering and economic benefits. This implies that the adoption of these technologies in the construction sector will significantly boost the country’s economy. According to the findings, training and educating professionals about the overall concept of IoT, as well as other design professionals, on the importance of using IoT elements, will aid in the level of adoption. As a result, steps must be taken to mitigate the high cost of IoT devices and training.

## Data Availability

Data generated or analyzed during the study are available from the corresponding author on reasonable request.
